# Using Decision Trees as an Expert System for Clinical Decision Support for COVID-19

**DOI:** 10.2196/42540

**Published:** 2023-01-30

**Authors:** Dillon Chrimes

**Affiliations:** 1 School of Health Information Science Human and Social Development University of Victoria Victoria, BC Canada

**Keywords:** assessment tool, chatbot, clinical decision support, COVID-19, decision tree, digital health tool, framework, health informatics, health intervention, prototype

## Abstract

COVID-19 has impacted billions of people and health care systems globally. However, there is currently no publicly available chatbot for patients and care providers to determine the potential severity of a COVID-19 infection or the possible biological system responses and comorbidities that can contribute to the development of severe cases of COVID-19. This preliminary investigation assesses this lack of a COVID-19 case-by-case chatbot into consideration when building a decision tree with binary classification that was stratified by age and body system, viral infection, comorbidities, and any manifestations. After reviewing the relevant literature, a decision tree was constructed using a suite of tools to build a stratified framework for a chatbot application and interaction with users. A total of 212 nodes were established that were stratified from lung to heart conditions along body systems, medical conditions, comorbidities, and relevant manifestations described in the literature. This resulted in a possible 63,360 scenarios, offering a method toward understanding the data needed to validate the decision tree and highlighting the complicated nature of severe cases of COVID-19. The decision tree confirms that stratification of the viral infection with the body system while incorporating comorbidities and manifestations strengthens the framework. Despite limitations of a viable clinical decision tree for COVID-19 cases, this prototype application provides insight into the type of data required for effective decision support.

## Introduction

COVID-19 presents a major and urgent threat to global health. Since the original outbreak of the disease in early December 2019 in Wuhan, China, COVID-19 has spread to over 188 countries, with the number of cases recently estimated at ~50-100 million and the number of fatalities at ~1-2 million, putting the death rate at approximately 3% [1-4]. In Canada, 30-40% of Canadians have contracted COVID-19, and the proportion of the infected population who experience severe illness is estimated at ~10% [5]. Clinicians dealing with this disease would benefit from an expert tool to rapidly assess severe COVID-19 cases.

The sources and modes of transmission of COVID-19 remain unpredictable [1], despite well-established scientific knowledge of the biology of the virus. COVID-19 is highly contagious, with a transmission capacity far greater than that of the previous severe acute respiratory syndrome (SARS) epidemic in 2003, and the virus reaches an extremely high abundance in infected people (up to 1 billion RNA copies per milliliter of sputum) [6]. Moreover, SARS-CoV-2 is stable on plastic and steel objects, where viable virus can remain detectable for more than 72 hours [6]. The wide, rapid spread of COVID-19, together with the population-level mortality rate have occasioned substantial research on its physiological and health effects, including its manifestations (ie, increasing effects on human biological functions) in conjunction with existing morbidities [1]. This formative study aimed at making accurate causal inferences with a view toward predictive modeling. Yet, the physiological interaction between COVID-19 and the human body is not yet well-established. Therefore, research models need to be reframed or even reinvented based on an assessment of the problems and strengths of targeting COVID-19, which can be achieved by using several intersecting knowledge bases concerned with human pathophysiology.

Mild cases of COVID-19 are typically characterized by early viral clearance, with 90% of mildly affected patients repeatedly testing negative on reverse transcriptase-polymerase chain reaction tests by day 10 postonset [7]. In contrast, all severe cases were still evaluated as positive for COVID-19 at or beyond day 10 postonset, with the median duration of viral shedding in survivors being 20 days, during which time the affected individuals are highly contagious [8]. In one study, the longest observed duration of viral shedding in survivors was 37 days [9]; other studies reported even longer durations [10]. Modeling of COVID-19 suggests that severe cases can result in chronic respiratory and cardiovascular conditions lasting months and even longer, with recoveries remaining only partial without treatment [7,8]. Therefore, an important aspect of diagnosis is the ability to determine if a case will be mild or severe, especially since—for patients with slow recovery—mild cases can become severe. Conversely, if a case is quickly diagnosed as mild, then the risk of intubation and mechanical ventilation causing further chronic respiratory problems could be minimized [11].

Current scientific research in the epidemiology of COVID-19, as well as public health surveillance, relies on modeling COVID-19 infections to predict outbreaks [1]. One systematic review of over 100 models of COVID-19 found that none of these models was successful either in modeling the outbreak or in predicting recovery versus mortality [8]. Recent studies have used medical images and radiology techniques to develop prognostic and diagnostic models for detecting COVID-19 using applied statistics and machine learning, and a “COVID-19 vulnerability index” was developed related to hospital admissions [7,12]. Radiology can quickly detect COVID-19 as a respiratory disease at first onset and offer a quick indication of COVID-19 severity; however, it does not have a high success rate in detecting severe cases within the important 10-day latency-to-replication phase of this virus [13,14]. Another potential approach is to use blood samples to verify cases of severe COVID-19, without needing X-rays or other tissue samples or biopsy [15]. Because uncertainties remain and patients’ responses to infection vary greatly—and because there is no cure yet available for this disease—we need new methods to detect severe cases accurately and quickly.

Epidemiologists and other health professionals are currently working together to model risks using applied Bayes theorem with machine-learning techniques. This approach promises to produce models that can accurately assess the probability of severe cases within a given infected population, as well as complications of cardiovascular diseases [8,16]. Although updates on available treatments are frequent, gaps in the clinical guidelines for treating COVID-19 remain [8], with few validated clinical decision-support tools [17] or clinical guidelines in constructing chatbots for COVID-19 (Table 1).

As the pandemic continues, clinicians need reliable information on human pathophysiological responses to COVID-19. Models commonly applied in prognosis and treatment include the simple nomogram (ie, a diagram between variables such as age, gender, renal function, medication dosage, and body weight), decision trees, score systems, and online tools that provide statistics on a range of metrics: in-hospital deaths, prolonged mechanical ventilation needs, and a composite of poor outcomes [18]. While there is little agreement as to which modeling techniques are useful in a hospital setting [8,19], studies seem to have found value in decision trees. One study of recovery in severe COVID-19 cases, based on testing of blood samples, showed that decision trees using Gini coefficients are more effective than models using support vector machines, logistic regression, or random forest classification [15]. Other studies indicate that decision trees can define complex outcomes of COVID-19, especially for severe in-hospital cases [20]. Thus, decision trees can be valuable to incorporate knowledge structures that support the effective treatment of severe COVID-19.

Recently verified predictive models used specific parameters as predictors in the diagnosis and prognosis of COVID-19: age, body temperature, lymphocyte count, and data obtained through lung imaging [19]. In addition, flu-like symptoms and neutrophil count are frequently used as predictors in diagnosis, while comorbidities, gender, presence of creatinine-reactive protein (CRP), and overall amount of creatinine are frequently used in prognosis [19]. Findings regarding the biology and differentiation of lung inflammation suggest that excessive quantities of cytokines result in inner (microvascular) and outer (macrovascular) heart problems and elevated risk of mortality [16]. Therefore, the main diagnostic-to-prognostic problem is the level of integration of COVID-19 with human biology and the variable degree of immune response. That is, some immune responses are beneficial in the body’s fight against COVID-19, while others are detrimental [21]. The diagnostic problem of ascertaining the probabilities of mortality and recovery can be simplified using binary classification, allowing for a variety of recovery-versus-death scenarios. The problem here is that there may be a variety of manifestations, each with its own specific physiological sequence, which can severely impact the body’s lung or heart [22]. Therefore, the design of a clinical decision-support tool to diagnose severe cases of COVID-19 would have to consider both these manifestations and any remaining gaps in that understanding.

The knowledge of COVID-19 manifestations is complex, since a range of chronic conditions are associated with lung, cardiovascular, and gastrointestinal diseases. One study showed that preceding coronavirus outbreaks such as SARS and Middle East respiratory syndrome (MERS) were associated with a significant burden of cardiovascular comorbidities [23]. Furthermore, diagnostic workups during SARS infections revealed detailed changes in electrocardiographic results, subclinical left ventricular diastolic impairment, and troponin elevation, all of which varied widely among patients [24]. Therefore, in the context of severe COVID-19 infection, any tools designed to support clinical decision-making need to take into account the interconnectedness of the body’s respiratory and cardiovascular systems. This accounting for interconnectedness can be accomplished by decision trees, which can synthesize knowledge structures in the architectural construction of their branches and leaves.

**Table 1:** Environmental scan of chatbots used for COVID-19 information.

Chatbot	Ages	Description	Setting	Inference method	Intervention effectiveness	Guidelines used	Prescription
Apple and Siri	All	Apple and Siri to help people who ask if they have the coronavirus	Anywhere	Uses data from Johns Hopkins University; rules-based	Only symptom-based	No clinical guidelines	No
Intermountain Healthcare	All	COVID-19 symptom checker	Research, primary care, and acute care in Utah at Intermountain Healthcare	Uses data from acute-care settings; rules-based	Effective to separate mild and severe cases based on symptoms	Simple clinical guidelines	Unknown
Google Dialogflow and Google Assistant	All	Chatbot designed with extensive prompts to entities and intents	Anywhere	Prototype, fact checker, and Q&A^a^ for COVID-19	Answers a variety of questions, including data on COVID-19 and symptoms	No clinical guidelines	No
COVIDradar	All	Chatbot and app to track health status	Anywhere within the United Kingdom and National Health Service	Prototype, fact checker, and Q&A for COVID-19	Effective to separate mild and severe cases based on symptoms and daily updates	Simple clinical guidelines	Unknown
Facebook Messenger with WHO^b^	All	COVID-19 fact and symptom checker	Anywhere	Prototype, fact checker, and Q&A for COVID-19	Answers a variety of questions, including data on COVID-19 and symptoms	WHO guidelines	No
BC CDC^c^	All	CDSS^d^-generated care suggestions based on agreed guidelines. These include what to do if testing negative for COVID-19	Research, hospital-based academic groups, possibly including clinicians	Prototype, fact checker, and Q&A for COVID-19	Answers a variety of questions, including data on COVID-19 and symptoms	Simple clinical guidelines	No

^a^Q&A: question and answer.

^b^WHO: World Health Organization.

^c^BC CDC: British Columbia Centre for Disease Control.

^d^CDSS: clinical decision support system.

## Design

Decision trees can incorporate medical knowledge [25], including human physiological responses to influenza and other diseases [20]. Human immune responses to COVID-19 can reveal a normal increase in T cells due to inflammation; however, this leads to a subsequent cytokine storm that increases the risk of mortality rather than reducing it. T-cell inflammation as a biological immune response to COVID-19 makes construction of the diagnostic decision branches in the knowledge tree difficult, requiring the introduction of additional calculations such as Gini coefficient thresholds [23]. The knowledge tree also needs to include a transition from diagnosis of chronic lung-to-heart conditions in the tissues, vessels, muscles, and valves. While the stratification of interactions between COVID-19 and chronic lung conditions and heart disease is not yet well understood [26], we have nonetheless been able to draw on existing clinical guidelines to construct the framework of a decision-support tool that models the transition from mild to severe COVID-19 cases.

**Figure 1:**
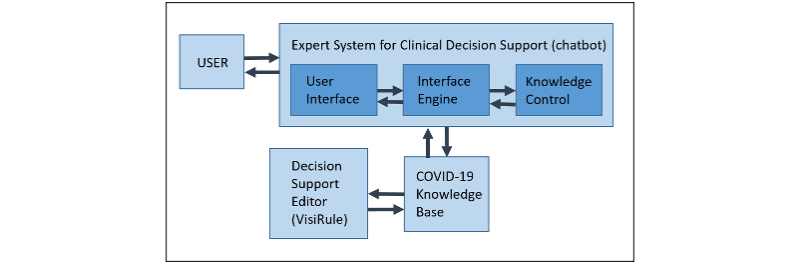
Expert system of a COVID-19 decision support web-based (chatbot) tool. There is an important interaction between the knowledge base (controlled) and an interface to display the chatbot to the user with a sequence of questions linked to the stratification of the COVID-19 disease course on an individual basis.

**Figure 2:**
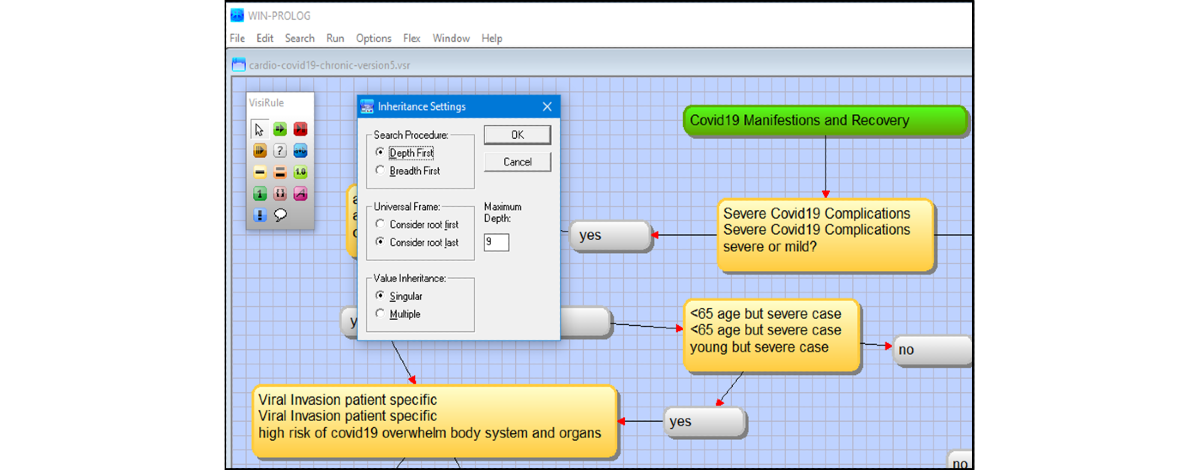
Screenshot from VisiRule 7.021. VisiRule inheritance settings, set as singular “Depth First” with a maximum of 9 from the root node. These settings are important as the complexity of the decision tree framework can be enhanced with the VisiRule applications and its PROLOG backend program.

**Figure 3:**
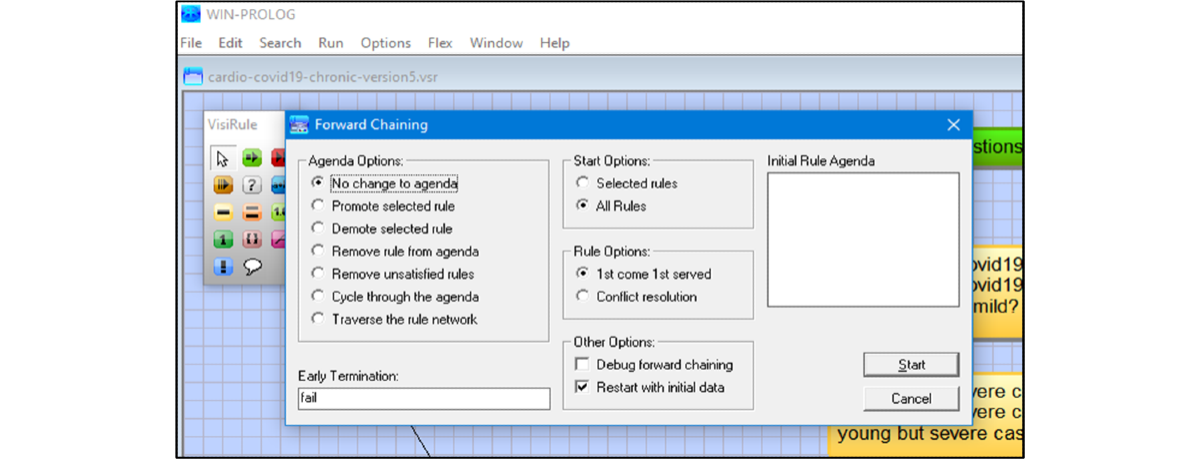
Screenshot from VisiRule 7.021 showing the forward chaining settings. Forward chaining is the default in this application for decision tree induction. As the pop-up display indicates, there are many more settings and customizations to add to the complexity of the forward chaining or traversing through the decision tree framework based on user input across the stratification.

After reviewing expert systems and other modeling technologies that use decision trees, we decided to use the infrastructure of a chatbot tool [27] that would either design decision trees manually or use Predictive Model Markup Language (PMML) schematic formats to create them. PMML can be utilized in tools such as KNIME and RapidMiner to automate the formation of a decision tree; in such procedures, data sets and trained data can be employed iteratively. In clinical applications, PMML has been applied to binary classification such as wound care management [27]. The graphical artificial intelligence software VisiRule has knowledge engineering, interface, and control/editor tools (Figure 1) with embedded inductive techniques with inheritance settings, set as singular “Depth First” with maximum of 9 from the root node (Figure 2) and forward chaining settings (Figure 3).

Induction (as well as deduction) can be used in the diagnosis of COVID-19. For example, the landmark decision tree program called c4.5 algorithm is a machine-learning workhorse that in its sequence of decision points can establish decision endpoints for classification [28]. Inductive and deductive reasoning can also be employed to modify the construction of the architecture of decision trees.

We attempted to create a diagnostic tool that could develop a decision tree based on existing COVID-19 data sets using a number of applications: Google’s Dialogflow, ZenChat, and KNIME. Dialogflow—a decision-support tool in the form of a chatbot—contains intents and entities and a knowledge base; its construction enables easy integration into web-based applications [29]. However, Dialogflow does not allow for binary classification; all it can do is enable a series of prompts in a specific sequence of COVID-19 queries. This makes modeling severe cases of COVID-19 extremely difficult, as we had no ability to incorporate Bayes theorem. Dialogflow thus could not be used to develop our planned chatbot. Another tool we considered, ZenChat, proved similar to Dialogflow, as it had no apparent capacity to generate decision trees using binary classification [30]. In the decision-tree framework in KNIME, which autogenerates a PMML schema, we used data sets on COVID-19 cases drawn from the website Kaggle.com. However, these COVID-19 data sets were not easily formed to binary classifications; notably, the data could not be easily sorted in terms of recovery, short or long recovery with onset of chronic conditions, or even risk of mortality. This difficulty in forming binary classifications was confirmed by running Bayes modeling of the data set in the Microsoft Azure Machine Learning Studio, which did not produce accurate results. Accordingly, we constructed the decision tree to account for health outcomes, stratified by comorbidities in the transition from the lung to the heart, while establishing binary classifications to manually calculate Bayes probabilities for each leaf endpoint (Table 2).

With the VisiRule construct (via upload of a .vsr file), prototypes were developed that allowed the user to trace the response in the decision tree. The maximum run was approximately 10-12 leaves, with an endpoint indicating a high risk of mortality, low risk of mortality with risk of morbidity, or prolonged recovery from COVID-19 [31]. These prototypes generated a report after each iteration of a user selecting “yes” or “no” in a set of questions linked to a patient’s health and biological responses. Results were also obtainable in other forms such as an HTML application that generated a list of results and a chatbot. Upon testing, it became clear that the decision tree used biological and physiological knowledge in deductive reasoning, with some inductive reasoning in the knowledge acquisition. However, rebalancing the decision tree (ie, user response to flow from the top node to leaf nodes that indicate the status of a person’s health with severe COVID-19 symptoms) will require additional data sets to allow for reordering the sequence of prompts, reducing or increasing the number of leaves leading to a decision point, and resampling data [25,32]. Furthermore, inductive, or inferential, reasoning is the process of moving from concrete examples to general models; that is, of learning to classify objects by analyzing a set of instances (eg, cases of illness that have already been resolved) whose classes are known [32].

In a previous study, data mining models were developed for the prediction of COVID-19 patients’ recovery using an epidemiological data set of COVID-19 patients in South Korea [23]. In that study, a decision tree, a support vector machine, naïve Bayes classifiers, logistic regression, random forest, and a K-nearest neighbor algorithm were applied directly to the data set and a model was developed using Python. The model predicted the age ranges of patients who are at elevated risk of dying from COVID-19, of those who are likely to recover, and of those likely to recover rapidly [23]. The results show that a model developed with a decision-tree algorithm can be most efficient in predicting the probability of recovery for COVID-19 patients.

To improve the tool’s inductive power, we included a set of lung conditions that can exacerbate cases of COVID-19 in the upper stratification of the decision tree: asthma, pneumonia, and chronic obstructive pulmonary disease [11]. In the next stratification, we incorporated elevated long-term risk of cardiovascular disease and hyperlipoidemia responses, because these have been linked with acute complications of COVID-19 [33].

**Table 2:** Stratified approach to the decision tree hierarchy for severe COVID-19 cases.

Stratification level	Sequence	Description
1	Viral invasion	Suggestive of the level of SARS-CoV-2 invading the cytoplasm of cells and binding to ACE2^a^
2	Age and body systems	Suggestive of higher risk of being aged ≥65 years with impacts on the respiratory and circulatory systems
3	Comorbidities	Suggestive of underlying chronic diseases
4	Manifestations	Suggestive of the level of viral infection and the human body’s responses

^a^ACE2: angiotensin-converting enzyme 2

## Objectives

The strength of clinical decision-support tools relies on the difficulty of detecting COVID-19 early in its interaction with human biological systems. Because of the high percentage of mild and asymptomatic cases, several pathophysiological responses to the disease need to be fully documented for risk of severity to be adopted [8,19]. Assessing severity, for instance, requires a prominent level of diagnostic accuracy, together with monitoring and periodic reassessment [17,26], all of which are more easily accomplished with the use of a decision-tree tool. In this study, the main objective of the decision-support tool was to diagnose severe cases of COVID-19 based on knowledge using VisiRule control/editor tools (Figure 1). Decision support in both diagnosis and prognosis takes into account a variety of signs and symptoms of COVID-19 on the one hand and the ability of human biological responses to combat the virus on the other hand. This main objective can be broken down into the following subobjectives:

1. Minimize the severity of COVID-19 through early detection via binary classification.
2. Assess the severity of cases with the extent of respiratory and cardiovascular involvement.
3. Reduce inaccuracies in the diagnosis of COVID-19, including both false negatives and false positives calculated by Bayes theorem.
4. Assess the risk of prolonged recovery, morbidity, and mortality.

In the process of achieving these subobjectives, a clear set of clinical guidelines for dealing with COVID-19 will be achieved.

**Figure 4:**
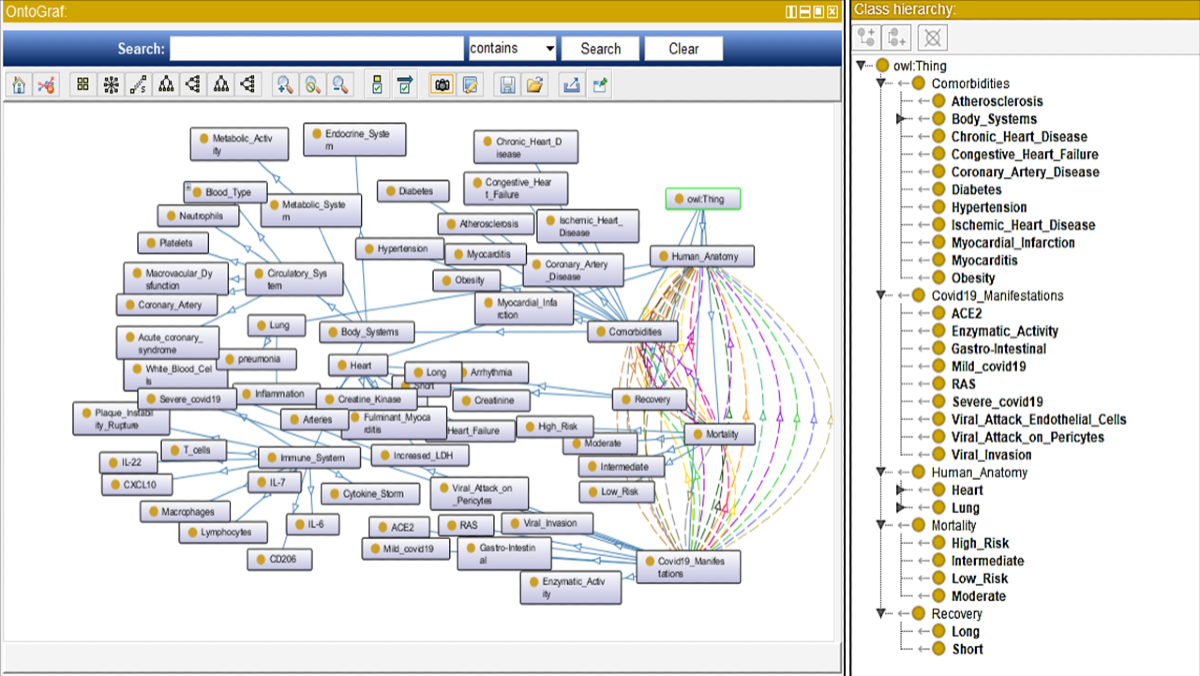
List of parameters for a decision-support (expert system) tool developed using Protégé 5.5.0. This image was extracted from the application showing the mapped stratifications of comorbidities, manifestations, heart and lung, and mortality and recovery.

**Figure 5:**
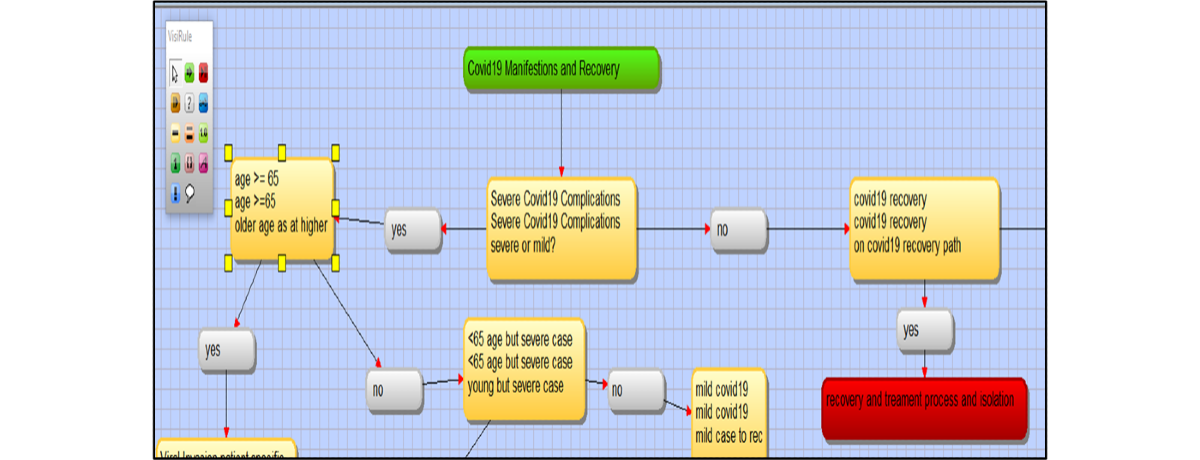
Screenshot from VisiRule 7.021 showing the separation of mild and severe COVID-19 cases. This display shows the start (in green), the question prompts (in yellow), and endpoint (in red).

In the diagnosis and treatment of COVID-19, an effective clinical decision-support tool would ensure that best practices are followed (refer to Figure 4, Table 2, and Table 3). Among the benefits provided by a clinical decision-support tool are: (1) incorporating health outcomes of severe COVID-19 cases; (2) considering respiratory and cardiovascular symptoms related to severe COVID-19; (3) correlating COVID-19 infection with other indicators such as obesity, diabetes, blood type, age, and heart and lung complications and illnesses; (4) covering a wide gamut of human phytopathology issues relevant to severe COVID-19; (5) separating mild COVID-19 cases from severe COVID-19 cases (Figure 5); and (6) predicting the risk of mortality.

The diagnostic and risk stratification used in a clinical decision-support tool can also be updated as knowledge of health outcomes and treatments are validated.

**Table 3:** Risk factors for severe cases of COVID-19.

Risk status			Relative risk (%)
**High risk**			
	Personal history of multiple comorbidities, including hypertension	35	
	Previous lung cancer or chronic pneumonia	17	
	Immune deficiencies	5 to 15	
	Personal history of cardiovascular diseases	9 to 10	
	History of heart issues such as arrhythmia	8	
	Immunosuppression	6 to 8	
**Moderate risk**			
	Asthma and chronic pulmonary obstructive disease	4.9 to 7.3	
	Slight inflammation	3.0 to 5.4	
	Issues with coronary artery and microvessels	5.4	
**Intermediate risk**			
	Asthma without chronic conditions but could have pneumonia or bronchitis	3.8	
	Asthma, hypertension, and slight inflammation	3.0	
	Age <65 years with microvessel and coronary artery issues	2.2	
**Low risk**			
	Age <65 years	0.5	
	Normal level of SARS-CoV-2 replication for 10 days and testing negative for COVID-19	0	

## Decision Tree Architecture

### Overall Design

Architectural construction of the decision tree had 212 yes/no questions, which integrate a range of immune, cardiovascular, and other biological responses to COVID-19 infection. We ran a script to generate a truth table with 990 rows and 64 columns, giving us 63,360 possible combinations of contracted COVID-19 cases. It has 48 end points in total, including 18 long recovery, 13 deaths, 10 chronic disease onsets with recovery, and 7 recoveries. The main knowledge details of the 959 lines of code are described below.

### COVID-19 and the Lungs

The host receptor through which SARS-CoV-2 enters cells to trigger infection is angiotensin-converting enzyme 2 (ACE2), which is expressed in the lungs, heart, and blood vessels. This process facilitates entry of the virus into the alveolar epithelial cells within the cytoplasm of the host’s skin. The viral RNA then starts to replicate, followed by viral shedding, which likely plays a pathogenic role, resulting in severe cases of lung injury and respiratory failure [33].

### COVID-19 and the Heart

**Figure 6:**
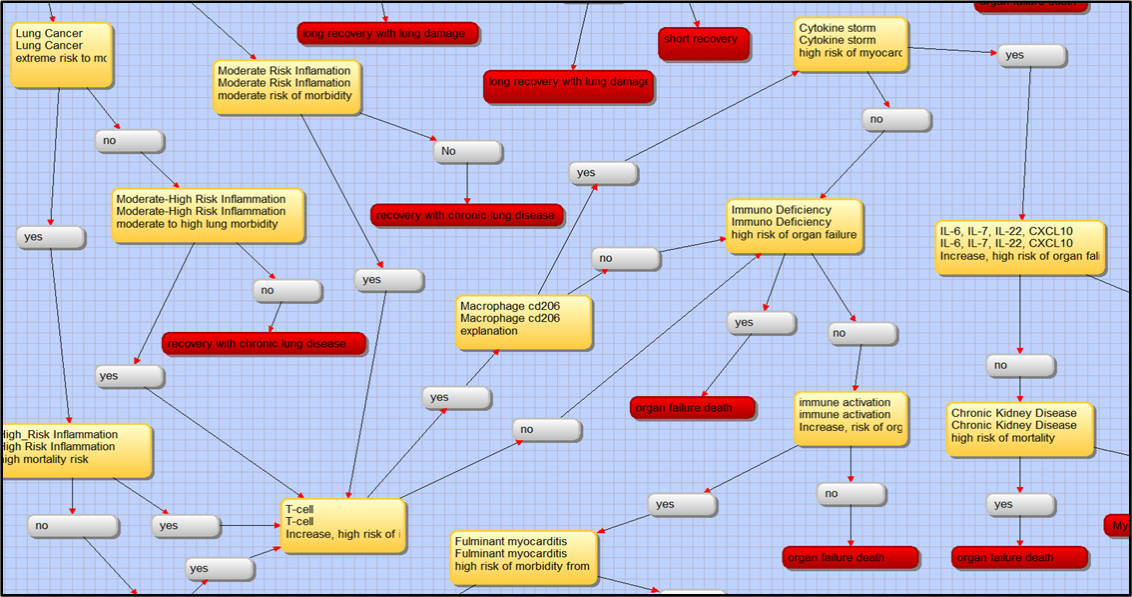
Screenshot from VisiRule 7.021 showing lung inflammation to T-cell activation and cytokine storm. This display shows the transition of question prompts (in yellow) from lung to heart conditions, including moderate to high risk, inflammation to immune deficiency, cytokine storm, and chronic kidney disease with binary responses (yes or no) toward the endpoint (in red).

COVID-19 is primarily a respiratory disease, but many patients also develop cardiovascular disease, including hypertension that can exacerbate the effects of COVID-19 [34-37], and acute cardiac injury [17]. ACE2 is highly expressed in the human heart, blood vessels, and gastrointestinal tract [17]; when COVID-19 infection dysregulates the ACE2 system, we see cardiovascular disease as the result. In a study of 416 COVID-19 patients, 57 of whom died of the illness, Shi et al [38] found that cardiac injury was common. Therefore, cardiac status and influences of COVID-19 should be part of the decision tree to determine the severity of the potential effects of COVID-19 on that person or patient. COVID-19 infections are also likely associated with infection-induced myocarditis and ischemia [36]. Elevated troponin T levels due to cardiac injury have been associated with a significant rise in mortality [33-37]. A cytokine storm, resulting from a combination of T-cell activation and dysregulated release of interleukin (IL)-6, IL-17, and other cytokines, may also contribute to cardiovascular disease in COVID-19 cases [17]. It is possible that activated T cells and macrophages may infiltrate the infected myocardium, resulting in the development of fulminant myocarditis and severe cardiac damage (Figure 6) [35,36]. This condition eventuality will also be fully integrated in the decision-support tool, with several linkages in the decision tree between cytokine storms, arrhythmias, microvascular dysfunction, and acute coronary syndrome (Figure 6) [35]. Cardiovascular disease may be a primary phenomenon in COVID-19, but it may also be secondary to acute lung injury, which increases the cardiac workload, a condition that is especially problematic in patients with congestive heart failure [21].

### COVID-19 and Comorbidities

Comorbidities identified in COVID-19 studies include chronic cardiac disease, chronic respiratory disease, chronic renal disease (estimated glomerular filtration rate ≤30), mild to severe liver disease, dementia, chronic neurological conditions, connective tissue disease, diabetes mellitus, and various malignancies [2,26]. Clinician-defined obesity is also classified as a comorbidity owing to its probable association with adverse outcomes in patients with COVID-19 in New York City [26].

### COVID-19 and the Immune System

Immune system activation may result in plaque instability, increasing the risk of acute coronary events such as stroke [21]. Additionally, research indicates that COVID-19 positivity is associated with lymphopenia (ie, abnormally low levels of white cells in the blood), damage to the liver and muscle tissue, and significantly increased levels of CRP [39,40].

## Limitations and Future Work

All of this knowledge from the research literature was incorporated into the flow of questions in the decision tree to form the COVID-19 clinical decision-support tool. However, there are a number of smaller limitations that limit the usefulness of the present design. Gender was not included as a variable in the decision tree; however, the effects of COVID-19 are covered in biological responses with underlying health issues of hypertension and coronary heart disease. COVID-19 patients are more likely to be male than female, and to have more comorbidities such as hypertension and coronary heart disease [9,24,41]. Men have a higher risk of cardiovascular complications than women. In addition, no geographical data were included and no sensitivity analysis was applied.

Another limitation of our work is that the decision-support tool was not based on data that we collected; rather, we made decisions for its construction based on research published during the pandemic. Information about the impact of COVID-19 on the human body is rapidly changing as the pandemic unfolds. For example, new findings indicate that the blood tests used in diagnosing congestive heart failure may also help to indicate severe cases of COVID-19 [39].

The decision tree is not considered to be very deep and would take less than 1 minute for a user to derive a result, status, or event based on certain health outcomes. I conducted stratification of 6-7 leaves to include the relevant parameters for the diagnosis and prognosis of COVID-19 [25]. However, the decision tree used in this decision-support tool did not set a threshold on the number of leaves per node, and instead added knowledge structures of COVID-19 with human anatomy and biology.

The use of clinical decision-support tools that incorporate decision trees is subject to an inherent limitation: the lack of circular references. Best practice for the diagnosis of severe cases of COVID-19 with comorbidities requires circular references and feedback loops in a system-dynamic approach, especially regarding pathophysiology. However, circular references and feedback loops are not possible in decision trees, which makes the application less dynamic to a real-time diagnosis.

The question remains: how would this decision tree framework and use of chatbot to assess COVID-19 integrate within the clinical setting? The answer relies on the inductive power of the chatbot to improve the sequence of the stratification of the level of viral infection, which can be assessed in a clinical setting [1], and the biological responses such as risk assessment of the human cardiovascular system (lung to heart), as Guzik et al [16] stated that stratification from COVID-19 severity increases from the lung to the heart. Furthermore, Knight et al [19] stated that no model can predict the outbreak and spread of COVID-19 in the population; however, there are indications on the duration of COVID-19 infection in individuals of populations that can range from mild to severe. Therefore, a decision tree framework that stratifies the impact of COVID-19 (on an individual basis) can contribute to a clinical setting in real time dealing with patients in the hospital compared to running reports and models (eg, “COVID-19 vulnerability index” related to hospital admissions [7,12]) that can be broadcasted to the public and influence policies such as travel, masks, and distance between people in a hospital rather than clinical patient care [13-19].

Another limitation is that the application of inference probabilities of Bayes theorem to the leaves of the decision tree were not interactive and static. There are uncertainties in the health outcomes of patients with severe COVID-19; therefore, the application of Bayes probabilities is important. Since we could not automate the construction of a decision tree from COVID-19 data, we had no method to automate a valid application of Bayes probabilities for the risk of mortality versus recovery. The structure of decision trees allows for knowledge acquisition and application of inference probabilities, although this framework cannot be clinically validated at this time.

Moreover, if the decision tree is primarily ontology-based on its binary classification, then the probabilities of COVID-19 could become more accurate and plausible based on medical conditions such as comorbidities. Khan et al [42] demonstrated a possible way to integrate a trained data set (using WEKA, MATLAB) and then integrate the data set with the ontology of relationships (via the Protégé application) to establish an ontology-based decision tree model. This method could be applied to our COVID-19 decision tree and could integrate ontology in its stratification, which would reduce the number of scenarios from 63,360 to a much lower number; the scenarios would have to incorporate ontology rules such as age and body system with comorbidities in a more succinct manner. In turn, a data set could be simulated and ratified toward the type of data that need to be collected to form a similar decision tree for accurate binary classification of stratified severe COVID-19 cases.

Finally, the use of decision trees makes it difficult to control for certain biases and overlapping. Decision trees use induction-to-deduction algorithms that range from traditional heuristic-based techniques to more recent hybrid data-to-tree approaches. These algorithms are essential in constructing a sequence of questions that flow from one to the next. For this reason, the basic features of the decision-support tool mitigate against the quantification of any inherent biases. For example, the stratification could be biased by the decision to design a sequence from chronic lung conditions to the heart instead of to gastrointestinal illnesses. This places an emphasis on knowledge of the cardiovascular system while ignoring underlying conditions affecting other body systems.

## Conclusion

In conclusion, a decision tree with stratification of COVID-19 effects on biological systems is important knowledge to prototype and simulate. Additionally, stratification of the human physiology within the decision tree proved to indicate that the questions of the health and status of the person with COVID-19 would result in an appropriate summary or list of conditions that are involved in clinical decision support in a specific sequence of events.

## Abbreviations

ACE2: angiotensin converting enzyme
CRP: creatine-reactive protein
IL: interleukin
MERS: Middle East Respiratory Syndrome
PMML: Predictive Model Markup Language
SARS: Severe Acute Respiratory Syndrome
